# InDuRanS: neoadjuvant immunochemotherapy followed by surgery or definitive chemoradiotherapy and consolidation durvalumab in resectable and borderline resectable stage IIB–IIIA/B non-small-cell lung cancer

**DOI:** 10.3389/fonc.2026.1909979

**Published:** 2026-10-07

**Authors:** Eleni Gkika, Cornelius F. Waller, Cas S. Dejonckheere, Julian P. Layer, Jan Meiners, Peter Brossart, Ernst Rodermann, Anne Sofie Schiefer, Marc Münter, Nils H. Nicolay, Anca L. Grosu, Andreas Rimner, Joachim Schmidt, Severin Schmid

**Affiliations:** 1 Department of Radiation Oncology, University of Bonn, University Hospital Bonn, Bonn, Germany; 2 Department of Hematology, Oncology & Stem Cell Transplantation, Freiburg University Medical Center, Faculty of Medicine, University of Freiburg, Freiburg, Germany; 3 Institute of Experimental Oncology, University of Bonn, University Hospital Bonn, Bonn, Germany; 4 AstraZeneca GmbH, Hamburg, Germany; 5 Medical Clinic III of Oncology, Hematology, Rheumatology, and Immune Oncology, University of Bonn, University Hospital Bonn, Bonn, Germany; 6 Praxis-Netzwerk Onkologie, Troisdorf, Germany; 7 Department of Radiation Oncology, Klinikum Stuttgart, Stuttgart, Germany; 8 Department of Radiation Oncology, University of Leipzig, University Hospital Leipzig, Leipzig, Germany; 9 Department of Radiation Oncology, University of Freiburg, University Medical Center Freiburg, Freiburg, Germany; 10 Department of Thoracic Surgery, University of Bonn, University Hospital Bonn, Bonn, Germany; 11 Department of Thoracic Surgery, University of Freiburg, University Medical Center Freiburg, Freiburg, Germany

**Keywords:** definitive chemoradiotherapy, durvalumab, induction immunochemotherapy, randomized trial, stage III NSCLC, surgery

## Abstract

**Background:**

The optimal integration of surgery and definitive chemoradiotherapy (CRT) following modern neoadjuvant immunochemotherapy in stage IIB–IIIA/B non-small-cell lung cancer (NSCLC) remains controversial. Although immune checkpoint inhibitors have substantially improved outcomes in both resectable and unresectable disease, prospective randomized data directly comparing surgery and definitive CRT after neoadjuvant immunochemotherapy are lacking.

**Methods:**

InDuRanS is a prospective, multicenter, randomized, open-label phase II trial conducted in Germany. Patients with (borderline) resectable stage IIB–IIIA/B (N2) NSCLC receive three cycles of neoadjuvant immunochemotherapy with durvalumab plus platinum–paclitaxel. Patients with persistent resectability are randomized 1:1 to surgery or definitive CRT. Both treatment arms receive consolidation durvalumab for up to 12 months. The primary endpoint is 2-year event-free survival in 158 randomized patients. Key secondary endpoints include progression-free survival, overall survival, safety and tolerability, health-related quality of life, and an extensive exploratory translational research program.

**Discussion:**

InDuRanS is the first randomized trial to directly compare surgery versus definitive CRT within a contemporary multimodal treatment sequence incorporating both neoadjuvant and consolidation immunotherapy in stage IIB–IIIA/B NSCLC. Its results are expected to help define optimal local treatment strategies in an evolving therapeutic landscape. InDuRanS is currently active and open to enrollment as of April 2025, with primary completion anticipated in 2028 (NCT06810609).

## Introduction

Stage IIB–IIIA/B non-small-cell lung cancer (NSCLC) represents a biologically and clinically heterogeneous disease with multimodal treatment as the standard of care (1). For patients with (potentially) resectable stage IIB–IIIA/B (N2 or ipsilateral mediastinal and/or subcarinal nodal involvement) disease, both curative surgery-based multimodality concepts and definitive chemoradiotherapy (CRT) are possible, although historical randomized trials have not demonstrated a clear survival advantage for either strategy. These trials (e.g., RTOG 8901, EORTC 08941, ESPATUE), however, predate modern minimally invasive staging and surgical approaches, advanced radiotherapy techniques, and the routine incorporation of immune checkpoint inhibitors (2–4).

The introduction of programmed death-1/programmed death-ligand 1 (PD-1/PD-L1) inhibitors has transformed the management of NSCLC. Durvalumab consolidation after definitive CRT has become standard treatment in patients with unresectable stage III disease (5, 6). In parallel, neoadjuvant and perioperative immunochemotherapy has demonstrated benefits in major pathological response and event-free survival (EFS) in those with resectable disease, in comparison with chemotherapy alone (7). Nevertheless, the optimal local treatment approach following neoadjuvant immunochemotherapy in operable stage IIB–IIIA/B disease remains undefined. The InDuRanS trial is designed to address this central question by directly randomizing patients to surgery or definitive CRT after standardized neoadjuvant immunochemotherapy in resectable and borderline resectable stage IIB–IIIA/B NSCLC.

## Methods

### Study design and population

InDuRanS (ARO-2024-12/AIO-TRK-0125/NCT06810609) is an investigator-initiated, prospective, multicenter, randomized, open-label phase II trial conducted at approximately 10 centers in Germany.

Eligible patients are adults with cytologically or histologically confirmed NSCLC, stage IIB–IIIA/B (N2) according to the ninth edition of the tumor-node-metastasis (TNM) classification (based on invasive mediastinal staging and positron emission tomography [PET]), deemed resectable or borderline resectable by interdisciplinary tumor board assessment. Eligibility requires that both surgery and definitive CRT are considered feasible local treatment options. Technical resectability is defined by the expectation that complete macroscopic and microscopic resection, including involved nodal disease, can be achieved, whereas borderline resectability denotes technically possible resection with an increased risk of incomplete resection. Where possible, the consensus definition of the multisocietal EORTC-Lung Cancer Group collaboration is followed (8). Functional operability is assessed according to cardiopulmonary reserve. Key inclusion criteria include Eastern Cooperative Oncology Group (ECOG) performance status 0–1, adequate cardiopulmonary reserve for multimodal therapy, and epidermal growth factor receptor (*EGFR*) and anaplastic lymphoma kinase (*ALK*) wild-type status (i.e., not eligible for approved molecular-targeted therapies). Key exclusion criteria include unresectable disease, N3 nodal involvement (i.e., contralateral mediastinal and/or hilar, deep cervical, or supraclavicular), distant metastases, active autoimmune disorders requiring systemic immunosuppression, prior immune checkpoint inhibitor therapy with relevant side effects, and significant comorbidities preventing safe delivery of protocol therapy. Key inclusion and exclusion criteria are summarized in **Table 1**.

**Table 1:** Key inclusion and exclusion criteria.

Inclusion criteria
Capable of giving written informed consent. Willing and able to comply with the trial protocol.
Age ≥18 years (no upper limit).
Histologically and/or cytologically proven NSCLC (*EGFR*m–, ALK–).
Stage IIB (one or more lymph node levels involved at EBUS/mediastinoscopy/PET/CT; T1 N2a) or stage IIIA (one or more lymph node levels involved at EBUS/ mediastinoscopy/PET/CT; T1 N2b or T2–3 N2a) or stage IIIB (one or more lymph node levels involved at EBUS/mediastinoscopy/PET/CT; T4 N2a or T2–T4 N2b).
ECOG performance status of 0 or 1.
Given or borderline technical/oncologic complete resectability (R0) at the time of inclusion with sufficient functional reserves for the planned surgery (fulfillment of adequate criteria for functional and medical resectability, stable cardiac function).
Adequate organ and bone marrow function.
Discussion in a multidisciplinary tumor board supporting participation in this clinical trial.
Before randomization
Given technical/oncologic complete resectability (R0) at the time of randomization.
Sufficient functional reserves for the planned surgery (if allocated).
Adequate organ and bone marrow function.

Exclusion criteria
Unresectable disease.
Mixed histology with areas of small cell carcinoma.
*EGFR*m or ALK+ disease.
Clinically symptomatic superior vena cava syndrome.
Diffuse mediastinal involvement.
Patients with N3 nodal involvement. Metastatic disease (M1).
Invasion of the thoracic aorta (T4 – aorta), heart (except for the left atrium; T4 – heart), esophagus (T4 – esophagus), or spine (T4 – spine). Note: T4 with invasion of the diaphragm is not excluded.
Pancoast tumor/superior sulcus syndrome (T3–4 Nx).
Endobronchial tumor extension to the contralateral main stem bronchus.
Lung or heart function not allowing the intended surgical procedure at the time of inclusion.
History of mediastinal irradiation.
In case of a history of anti-PD-1, anti-PD-L1, or anti-CTLA-4 therapy: toxicity that led to permanent discontinuation, ongoing adverse event, any grade 3+ immune- related adverse event (for neurologic or ocular adverse event: any grade).
Participation in another clinical trial with an investigational product during the last 12 months.
History of idiopathic pulmonary fibrosis, pneumonitis, organizing pneumonia, or evidence of active pneumonitis and other infections.
Active or prior documented autoimmune or inflammatory disorders (exceptions apply).
Major surgical procedure (as defined by the Investigator) within 28 days before the first dose of durvalumab.
History of another primary malignancy (exceptions apply) or leptomeningeal carcinomatosis.
History of allogeneic organ or stem cell transplantation. Primary immunodeficiency.
Insufficient patient compliance (e.g., symptomatic psychiatric disorder) or refusal to participate.
Before randomization
Any unresolved grade 2+ adverse event (exceptions apply).

NSCLC, non-small-cell lung cancer; *EGFR*, epidermal growth factor receptor; m–, not mutated; *ALK*, anaplastic lymphoma kinase; EBUS, endobronchial ultrasound; PET/CT, positron emission tomography/computed tomography; ECOG, Eastern Cooperative Oncology Group; m, mutated; PD-1, programmed death-1; PD-L1, programmed death-ligand 1; CTLA-4, cytotoxic T-lymphocyte-associated protein.

### Treatment regimens

All enrolled participants receive three 21-day cycles of neoadjuvant therapy with durvalumab (1,500 mg intravenously on day 1 of each cycle), paclitaxel (175 mg/m^2^ intravenously on day 1), and cisplatin (50 mg/m^2^ on days 1 and 8 of each cycle, or carboplatin at the investigator’s discretion).

Upon completion and following restaging, including mandatory PET/CT with PET Response Criteria in Solid Tumors (PERCIST) and Response Evaluation Criteria in Solid Tumors (RECIST; version 1.1) assessment (repeat invasive mediastinal staging is not routinely mandated and may be performed when clinically indicated), participants with persistent resectability will be randomized (1:1) to local treatment, stratified according to stage (IIB–IIIA versus IIIB) and PD-L1 status (<1% versus ≥1%):

- Arm A (surgery): Tailored lung resection (lobectomy is considered standard; bilobectomy and pneumonectomy are permitted when required to achieve complete resection) with complete radical hilar and mediastinal systematic lymph node dissection. The goal is complete oncological resection (R0), and intraoperative frozen section assessment is mandatory. If microscopic residual disease is identified, additional resection should be performed where technically and functionally appropriate. In patients with R+ resection, protocol-specified treatment continues, and postoperative radiotherapy is not permitted. For participants randomized to the surgery arm, all surgery-related events will be recorded from the date of surgery to 90 days thereafter, according to the Clavien–Dindo classification.
- Arm B (definitive CRT): PET-based intensity-modulated radiotherapy to a total dose of 60–70 Gy in fractions of 2 Gy, combined with two 21-day cycles of platinum (50 mg/m^2^ cisplatin on days 2 and 9, cycle 1; or carboplatin at the investigator’s discretion) and vinorelbine (20 mg/m^2^ bolus given on days 2 and 9, cycle 1) and platinum (40 mg/m^2^ cisplatin on day 2, cycle 2; or carboplatin at the investigator’s discretion) and vinorelbine (15 mg/m^2^ bolus given on days 2 and 9, cycle 2). The prescribed total radiation dose is selected according to normal tissue exposure. The protocol includes standardized target volume delineation and organ-at-risk (OAR) constraints. Furthermore, all treatment plans will be centrally reviewed after trial completion. Each participating center must successfully complete a contouring and planning dummy run before enrolling its first study participant.

Both treatment arms will receive consolidation durvalumab (1,500 mg intravenously every 4 weeks) for up to 13 cycles following completion of surgery or definitive CRT (**Figure 1**).

**Figure 1:**
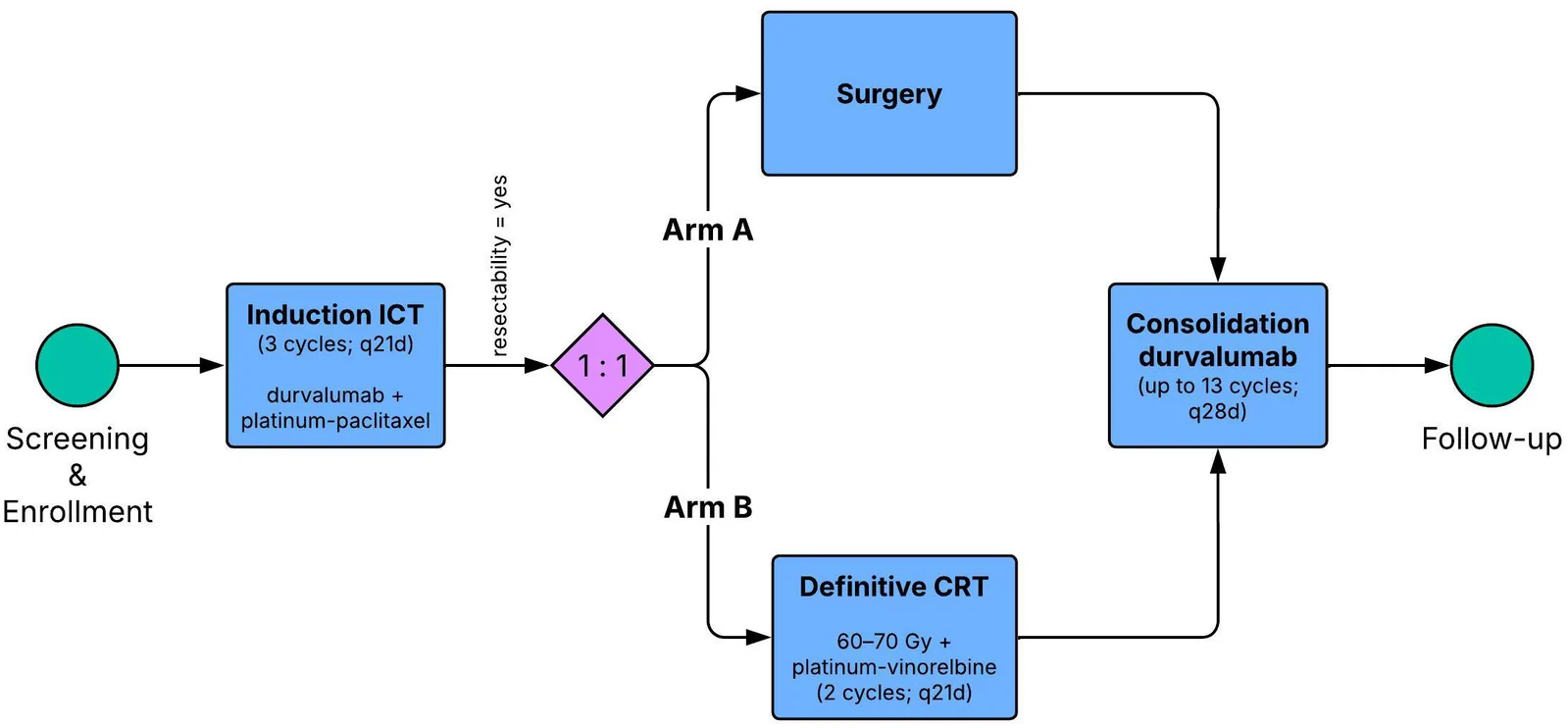
InDuRanS study design. ICT, immunochemotherapy; CRT, chemoradiotherapy.

In participants who do not fulfill the randomization criteria (e.g., those with progressive disease, prohibitive toxicity, loss of technical/oncological resectability and/or functional operability), protocol study treatment will be terminated and regular treatment continued at the discretion of the treating investigators. In all cases, protocol-defined safety follow-up will be performed.

### Endpoints and sample size

Cross-sectional imaging is performed after completion of local treatment, at 6 and 12 months after local therapy during consolidation treatment, and subsequently during follow-up. Follow-up visits are performed every 3 months through 24 months after randomization and thereafter every 6 months until the end of the study. Additional imaging is obtained when clinically indicated. Disease assessments are performed locally at the participating centers; no independent radiological central review is planned. The primary endpoint is 2-year EFS, defined per RECIST version 1.1 (for the assessment of measurable disease) as the time from randomization to disease progression (in non-resected participants), disease recurrence (after surgery), occurrence of a secondary malignancy, or death from any cause. The expected 2-year EFS in the surgical arm is approximately 63%, based on the results of CheckMate 816 with nivolumab-based neoadjuvant immunochemotherapy in resectable NSCLC (9). The trial has an estimation framework rather than a confirmatory hypothesis-testing framework: no formal superiority, non-inferiority, or equivalence hypothesis is tested. Sample size calculations considered 2-year EFS rates of 40%–60% in the CRT arm. With 158 randomized participants (79 per arm), the total width of the approximate 95% confidence interval for the between-arm difference in 2-year EFS proportions is approximately 30 percentage points across this range. Assuming 10% attrition before randomization, 176 participants will be enrolled. The primary analysis will follow the intention-to-treat principle and include all randomized participants according to randomized treatment allocation, irrespective of treatment actually received. Additional per-protocol and modified per-protocol analyses are planned. Two-year EFS will be estimated using Kaplan–Meier methods.

Secondary endpoints include progression-free survival (PFS), overall survival (OS), safety (including surgery-related events, recorded for 90 days after surgery according to the Clavien–Dindo classification, 30- and 90-day perioperative mortality, CRT- and immune-mediated toxicities with pneumonitis as an event of special interest), and tolerability of all study procedures (per Common Terminology Criteria for Adverse Events [CTCAE]), patterns of failure, and health-related quality of life measured with the European Organisation for Research and Treatment of Cancer (EORTC) Quality of Life Questionnaire (QLQ) C30 (core module) and LC13 (lung cancer module).

Exploratory translational endpoints will investigate biological tumor and tumor microenvironment features by longitudinal tissue and blood sampling, with a particular focus on systemic and inflammatory markers, plasma proteomics, circulating tumor DNA (ctDNA), peripheral immune cell phenotypes, tumor tissue expression profiles, and their correlation with clinical and radiographic outcomes. These analyses aim to identify biological correlates of response, resistance, safety, and treatment arm-specific effects, with a particular emphasis on early biomarkers and integrated multiparameter signatures for hypothesis generation and future risk stratification.

InDuRanS incorporates an extensive radiotherapy quality assurance (RTQA) program to promote protocol adherence and uniformity of radiotherapy delivery across centers, as rigorous RTQA has been associated with improved trial validity and reduced variability in patient outcomes (10).

### Ethics and regulatory aspects

This trial is conducted in accordance with the Declaration of Helsinki, International Council for Harmonization - Good Clinical Practice (ICH-GCP) guidelines, and applicable national regulations. Approval by institutional review boards and competent authorities was obtained before trial initiation. All participants will provide written informed consent before any study-specific procedures. InDuRanS is currently active and open to enrollment as of April 2025. As of August 2026, four patients have been enrolled and two patients have been randomized. Primary completion is anticipated in 2028.

## Discussion

An unresolved issue in stage IIB–IIIA/B (N2) NSCLC is the selection of the optimal local treatment strategy after neoadjuvant therapy. Historical randomized trials have not demonstrated a clear OS advantage of a surgery-based over a definitive radiotherapy-based multimodality strategy: in EORTC 08941, patients with stage IIIA (N2) NSCLC responding to neoadjuvant chemotherapy were randomized to surgical resection or radiotherapy (40% required postoperative radiotherapy), without a significant difference in PFS or OS (4). Similarly, INT 0139 (RTOG 9309) did not demonstrate a significant OS benefit for the addition of surgery to CRT (11). The available evidence suggests that both surgery-based multimodal approaches and definitive CRT yield comparable OS outcomes in this setting, as demonstrated in earlier comparative analyses (12). However, these data are largely derived from studies conducted before the widespread implementation of modern staging techniques, minimally invasive surgical procedures, and advanced radiotherapy technologies, thereby limiting their applicability to contemporary practice. Neoadjuvant immunotherapy in particular is emerging as a compelling strategy in locally advanced NSCLC by enhancing systemic antitumor immunity at a time of maximal tumor antigen exposure (13). Importantly, administering immune checkpoint inhibition before local treatment may prime and expand tumor-specific T-cell clones more effectively than adjuvant therapy alone, potentially translating into more durable disease control, thus increasingly redefining multimodal treatment concepts for patients with (borderline) resectable NSCLC (13). Although immune checkpoint inhibitors have been repeatedly tested in the perioperative setting, few trials have investigated their additional benefits as part of neoadjuvant immunochemotherapy before definitive chemoradiotherapy. Recently, the single-arm phase II APOLO trial integrated three cycles of atezolizumab with carboplatin and paclitaxel before standard concurrent chemoradiotherapy, followed by maintenance atezolizumab for up to 16 cycles (14). This approach showed an acceptable safety profile and encouraging EFS and OS rates, with nearly half of patients achieving partial responses after neoadjuvant therapy. The small sample size (*n* = 38), relatively short follow-up (median 30 months), and uncontrolled design do limit generalizability, however, and the findings warrant confirmation in larger trials. Additionally, the MDT-BRIDGE trial recently reported the feasibility of a durvalumab-based multimodal treatment approach incorporating an individualized local treatment allocation within a multidisciplinary framework. Importantly, this study supports the concept that treatment stratification based on response and resectability after neoadjuvant therapy is clinically implementable and may optimize patient selection for surgery versus definitive CRT (15). However, the non-randomized design and limited sample size preclude definitive conclusions regarding comparative efficacy between local treatment modalities at this time.

InDuRanS addresses a central unresolved question in the management of stage IIB–IIIA/B (N2) NSCLC: the optimal local treatment strategy following neoadjuvant immunochemotherapy. Although both surgery-based multimodality therapy and definitive CRT are established standards of care, these approaches have never been compared directly in a prospective randomized trial within the context of modern, immunotherapy-based treatment sequences. By incorporating neoadjuvant immunochemotherapy, randomized allocation to surgery or definitive CRT, and standardized consolidation durvalumab across both treatment arms, InDuRanS mirrors contemporary real-world practice and reduces confounding inherent to historical comparisons. Importantly, the strict inclusion of a homogeneous stage IIB–IIIA/B (N2) population enhances the internal validity and allows for a more meaningful comparison of local treatment strategies in a clinically common and well-defined subgroup. The trial’s comprehensive translational program further provides an opportunity to elucidate biological determinants of treatment response, resistance, and disease recurrence, including biomarkers associated with the depth and duration of treatment benefit.

Several limitations to the current design should be considered. First, randomization occurs only after completion of neoadjuvant immunochemotherapy and therefore selects patients who remain eligible for both local treatment approaches. Consequently, the randomized population differs from that in trials such as CheckMate 816 (on which the current sample size is based), in which randomization occurred *before* neoadjuvant therapy, and therefore represents a selected post-neoadjuvant population. Second, the phase II sample size was selected to provide preliminary comparative estimates with defined precision rather than to demonstrate superiority, non-inferiority, or equivalence. The results should primarily inform the design and assumptions of subsequent confirmatory trials. Finally, InDuRanS compares two complete multimodality strategies rather than the isolated effects of surgery or radiotherapy, since definitive CRT includes concurrent platinum–vinorelbine chemotherapy.

The findings of InDuRanS are anticipated to inform interdisciplinary decision-making for patients with operable stage IIB–IIIA/B NSCLC and may contribute to the refinement of future therapeutic algorithms and guideline recommendations. Ultimately, this trial aims to clarify the relative benefits and risks of competing local treatment strategies within an integrated immunotherapy framework, thereby strengthening the evidence base for durable and personalized care in this challenging disease stage.
